# Risk perception and mental health among college students in China during the COVID-19 pandemic: A moderated mediation model

**DOI:** 10.3389/fpsyt.2022.955093

**Published:** 2022-08-01

**Authors:** Ling Li, Hua Cao, Ling Yang, Changhu Yan, Xinru Wang, Yanhong Ma

**Affiliations:** School of Psychology, Northwest Normal University, Lanzhou, China

**Keywords:** coronavirus disease, risk perception, perceived stress, mental health, perceived control

## Abstract

Since the outbreak of the COVID-19 epidemic, it has spread on a large scale around the world, seriously affecting people’s physical and mental health. In China, almost all schools have postponed semesters, suspended offline classes, and implemented closed-off management, which has brought significant challenges to the study and life of college students. The study aimed to explore the relationship between risk perception, perceived stress, perceived control, and mental health among Chinese college students. This cross-sectional study was conducted among 1,856 college students. The results showed that risk perception was positively correlated with mental health. After adding the mediating variable of perceived stress, risk perception still significantly predicted mental health. In addition, the interaction term of perceived stress and perceived control significantly negatively predicted mental health. Specifically, perceived stress significantly affected mental health in the low-perceived control group. In contrast, in the high-perceived control group, the predictive effect of perceived stress on mental health disappeared. The present study showed that perceived stress partially mediated the relationship between risk perception and mental health; perceived control moderated the relationship between perceived stress and mental health, and high perceived control could buffer the effect of perceived stress on mental health.

## Introduction

The novel coronavirus disease (COVID-19) pandemic has spread rapidly all over the world, which has brought significant changes to the country, such as economic recession, corporate downtime, unemployment, and school closures. Due to the lethality, contagion, lack of specific treatment, and threat to the personal safety of this epidemic, it has posed a serious impact on people’s physical and mental health in crisis events (1). This is the worst large-scale public health emergency in China in recent years. Therefore, it is of great practical significance to construct and analyze the impact of public health emergencies on people’s mental health and coping behaviors in this context.

The college years have been found to be a particularly important time for lifespan development (2). For many people, college life should be a happy and exciting time. However, the reality of the rapid global spread of COVID-19 pandemic has brought many challenges for college students. The abrupt disruption of daily life, the cancelation of expected campus activities, the loss of social connections, and the change of learning styles have created a sense of threat, uncertainty, and stress (3). Unlike previous viral threats such as SARS, Ebola, and MERS, the COVID-19 pandemic has been more vividly presented in the massive and sustained global media coverage since the outbreak. Gao et al. (4) found that mental health problems were positively associated with frequent social media exposure during the COVID-19 outbreak. Therefore, college students exposed to mobile phones and the internet for a long time may have more mental health problems (5–7). Evidence suggested that college students experienced more psychological distress, manifested by higher levels of both anxiety and depressive symptoms than general workers during the pandemic (7). Ma et al. (5) found in an online survey of Chinese college students from 108 colleges and universities (*N* = 746,217) that about 45% of the participants had mental health problems, and about 35, 21, and 11% of the participants reported probable acute stress, depressive and anxiety symptoms, respectively.

Perceiving and avoiding risk are human instincts, which are adaptive evolution produced by humans. However, there is a substantial deviation between objectively present risk and subjective risk perception. Specifically, when faced with risk information, individuals often do not make risk assessments based on rationality, but make risk perceptions based on intuition (8). Risk perception refers to an individual’s intuitive feeling and understanding of various objective risks in the outside world, including the judgment of the possibility and potential hazards of crisis events (8). The risk resilience model suggest that risk factors and adverse environments can exacerbate adverse outcomes (e.g., fear, anxiety, depression) (9, 10). In this model, risk can be defined in different ways, including negative life events in recent months or in a lifetime, large-scale community trauma, adverse living environments, and cumulative risk calculations that combine these different types of risk factors (9). Wineman (11) believed that the direction and severity of the consequences of sudden public health crises were uncertain, and this uncertain risk perception would increase people’s psychological pressure and negative emotions, which seriously affects people’s mental health. This view had also been verified and research had found that risk perception was significantly associated with mental health (12, 13). For example, during the SARS and Ebola outbreaks, higher perceived risk was found to be associated with more significant mental health problems (13). Consistent results were also found in COVID-19 disease, that the severity of perceived risk was associated with poor mental health (14, 15). Sloan et al. (15) found that risk perception (e.g., fear of contracting COVID-19) was associated with poor mental health. Liu et al. (14) also found that higher risk perception was significantly associated with greater depressive symptoms in the Chinese population. During the COVID-19 pandemic, high levels of risk perception, such as fear of contracting the virus, might translate into serious mental health problems, including anxiety, depression, insomnia, and social withdrawal (16).

When a person feels threatened by a risk, stress is likely to occur (17). Faced with the same stressor, each person has different perceptions of stress due to their own experiences. Perceived stress refers to the psychological confusion or threat caused by various stimulating events and adverse factors, usually manifested as physical and mental tension and discomfort (18). Cohen et al. (19) considered that the impact of “objectively” stressful events depended to some extent on a person’s perception of stress. Within stress process theory, many stressful experiences don’t spring out of a vacuum. Negative life events may induce adverse changes in people’s lives and these adverse changes intensify the level of stress that people experience. Thus events create stress not only or even primarily through their direct demand for readjustment, but also through their indirect exacerbation role strains (20). Individuals would predict future outcomes by making comprehensive judgments on risk information in negative life events. If this outcome was full of uncertainty and harmfulness, stress would follow. It could be said that perceived stress mainly stemmed from a sense of threat and expectations of adverse future outcomes (21). The main feature of risk perception is the sense of threat caused by uncertainty (22). During the pandemic, unpredictable and threatening conditions such as these create perceived stress in college students. Although there was no relevant research on risk perception and perceived stress among college students, we have found a relationship between perceived stress and perceived risk in other groups (1, 23). For example, Li and Lyu (1) found that perceived stress was positively correlated with risk perception among the Chinese general public and the higher the level of risk perception, the greater the perceived stress of people. This may be because that perceived stress occurs when an individual feels inability to control the situation or manage emotional response to it (24). In addition, there is also an association between perceived stress and mental health (25–27). Perceived stress is an important risk factor for the low mental health of college students (25). For example, perceived stress was positively correlated with depression and anxiety (26) and negatively correlated with sleep quality (28). Stress process theory posits that social experiences translate into distinct health outcomes through exposure to stress and its coping ability (20). Risk perception brings perceived stress to college students, which may lead to negative psychological symptoms (29). Therefore, we speculated that perceived stress may mediate the relationship between epidemic risk perception and mental health.

The Stress-buffering Hypothesis states that an individual’s positive traits will mitigate the potential negative effects of perceived stress on psychological functioning and optimize event outcomes (30, 31). This may indicate that the relationship between perceived stress and mental health is moderated by other factors. As a positive trait, perceived control is a subjective perception of objective control, which refers to the belief that individuals can influence the progress of events through themselves, rather than external factors, and achieve the desired results (32). Individuals with high perceived control are able to take control of their lives, while individuals with low perceived control exhibit behavioral rigidity because they feel that the world cannot be changed (33). Previous researches had demonstrated an interrelationship between perceived stress and perceived control, with perceived control contributing to stress reduction (34). This may be because individuals with high perceived control have a strong sense of self-efficacy (35), and perceived control encourages individuals to adopt positive coping styles to solve problems, thereby reducing their perceived stress (36, 37). Enhanced perceived control can also improve health and life satisfaction (38). Researches had shown that perceived control was closely related to mental health (39–41). Enhanced perceived control helped to reduce the experience of stress and the risk for anxiety and depression (42, 43), and buffer the detrimental effect on subjective wellbeing (44, 45). Additionally, one study demonstrated that perceived control buffered the effects of stress on psychological distress (34, 46). The risk resilience model suggest that risk factors and adverse environments can exacerbate adverse outcomes (e.g., fear, anxiety, depression), and that individuals with adequate internal and external resources can offset the adverse outcomes of risk factors (9). According to the transactional model of stress (47), perceived control is an important internal (stress coping) resource for the individual. Accordingly, as perceived control decreases, college students who face high risk factors (perceived stress) experience a more negative mental health states. Specifically, compared with college students with high perceived control, the perceived stress of college students with low perceived control has a more serious negative impact on their mental health. Therefore, we speculated that perceived control would play a moderating role in the relationship between perceived stress and mental health.

**FIGURE 1:**
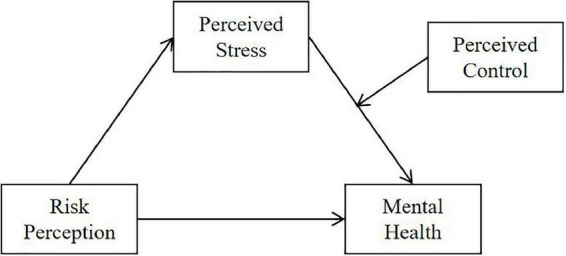
The expected moderated mediation model.

In conclusion, the study aim to investigate the mediating role of perceived stress in the relationship between risk perception and mental health, and examine the moderating role of perceived control in the relationship between perceived stress and mental health. Based on previous research, we proposed two hypotheses (see Figure 1):

Hypothesis 1: Perceived stress mediated the relationship between risk perception and mental health among Chinese college students during the COVID-19 pandemic.

Hypothesis 2: Perceived control moderated the relationship between perceived stress and mental health among Chinese college students during the COVID-19 pandemic.

## Materials and methods

### Participants and procedure of recruitment

Participants were recruited from 8 to 15 November in 2021 during the COVID19 pandemic, and all data were collected through self-report questionnaires in wjx.cn, a reliable Chinese online platform. A total of 1,856 final samples of college students from 15 provinces or autonomous areas in China were retained after excluding cases of invalid answers (e.g., continuous use of an option, too-short answering time), with a valid response rate of 97.7%. To protect participants’ privacy, data collection process was anonymous. The research team collected no personally identifiable information.

Among the participants, 86.7% were girls and 13.3% were boys, aged from 17 to 25 years (*M* = 20.1, *SD* = 1.6). The sample included 673 first-year students, 431 sophomores, 511 juniors, and 241 seniors. The study emphasized voluntary participation and did not provide participants with incentives. The ethical committee of the School of psychology, Northwest Normal University, approved this study before data collection.

### Measures

#### Mental health

Symptom checklist 90 [SCL-90; (48)] was used to measure participants’ mental health. This scale has 90 items rated on a 5-point Likert scale representing symptom severity, ranging from 1 (no symptom) to 5 (severe symptom). Total score range from 90 to 450, with higher scores indicating worse mental health. The scale has shown good validity and reliability in Chinese college students (49), and the Cronbach’s alpha in this study was 0.98.

#### Risk perception

The Perceived Risk of COVID-19 Pandemic Scale (PRCPS) was used to measure risk perception (50). This scale has 9 items, including the degree of worrying about contracting COVID-19 (not at all to very worried), the chance of contracting COVID-19 (zero to very high), and imagining yourself contracting COVID-19 (very difficult to very easy), etc. Total scores range from 9 to 47, with higher scores indicating greater risk perception. The Cronbach’s alpha in this study was 0.81.

#### Perceived stress

The Perceived Stress Scale-10 (PSS-10) was used to measure the extent of perceived stress over the past month (19). This scale has 10 items, rated on a 4-points Likert scale ranging from 0 (never) to 4 (very often). Total scores range from 0 to 40, with higher scores indicating greater perceived stress. The scale has shown good validity and reliability in Chinese college students (51), and the Cronbach’s alpha in this study was 0.88.

#### Perceived control

The Sense of Control Scale was used to measure perceived sense of control (40). This scale has 12 items rated on a 7-point Likert scale (1 = strongly disagree, 7 = strongly agree). Items include “I can do just about anything I really set my mind to” and “I often feel helpless in dealing with the problems of life” (reverse scoring). Total score range from 12 to 84, with higher scores indicating greater levels of perceived control. It has strong reliability (52), and the Cronbach’s alpha in this study was 0.73.

### Statistical analyses

Data analyses were performed with IBM SPSS 21.0 statistical software (IBM, Armonk, NY, United States) for Windows, and the significance level was set at *p* < 0.05 throughout the analyses. Firstly, we used the SPSS to calculated the descriptive statistics and the relationship among the variables by Pearson’s correlation analysis. Secondly, we used the SPSS macro PROCESS (Model 14) proposed by Hayes (53) to test the moderated mediation model. All regression coefficients were tested using the bias-corrected percentile bootstrap method. Bootstrapping (5,000 bootstraped samples) with 95% confidence intervals (CIs) was conducted to examine the significance of the mediation and moderation effects, and 95% CIs without zero indicated that the effects was significant. All model estimations were conducted with Mplus 8.3 (54) using maximum likelihood estimation. Monte Carlo power analyses suggested that the sample size was sufficiently large to detect small effects [i.e., 0.10 (55)] in moderated mediation model with power > 0.80 (all above 0.95, see Supplementary Table 1). In addition, we controlled for participants’ gender (0 = female, 1 = male) because it was reported to be related to individuals’ mental health (56).

## Results

### Common method deviation test

Harman Single-factor Test was used to test the common method deviation, and it was found that there were 12 factors with eigenvalues greater than 1. The first factor could explain 21.76% of the variation, which was less than the standard threshold value of 40%. This result indicated that there was no obvious common method deviation in this study.

### Descriptive statistics and correlation analyses

The descriptive statistics and correlations were presented in Table 1. Risk perception, mental health, perceived stress, and perceived control were found to be significantly correlated with each other. Epidemic risk perception was positively associated with mental health (*r* = 0.233, *p* < 0.01) and perceived stress (*r* = 0.132, *p* < 0.01), but negatively correlated with perceived control (*r* = −0.241, *p* < 0.01). Perceived stress was positively associated with mental health (*r* = 0.229, *p* < 0.01), but negatively correlated with perceived control (*r* = −0.185, *p* < 0.01). Perceived control was negatively correlated with mental health (*r* = −0.424, *p* < 0.01).

**TABLE 1:** Descriptive statistics and correlations.

Variables	*M*	*SD*	1	2	3	4	5
Risk perception	17.61	5.37	1				
Mental health	120.70	40.20	0.233**	1			
Perceived stress	18.72	6.53	0.132**	0.229**	1		
Perceived control	56.12	9.30	−0.241**	−0.424**	−0.185**	1	
Gender	0.13	0.33	−0.073*	0.021	−0.082*	–0.042	1

N = 1,856. *p < 0.05, **p < 0.01. Gender: 0, female; 1, male.

### Examination of moderated mediation model

**FIGURE 2:**
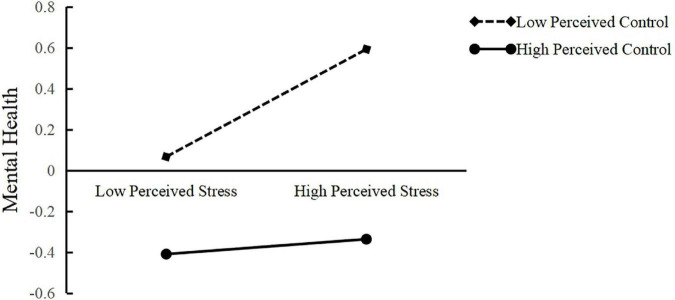
Perceived control moderates the relationship between perceived stress and mental health.

Table 2 showed the main results of our moderated mediation model. Model 1 examined the effect of risk perception on perceived stress, and Model 2 examined the effects of risk perception, perceived stress, and perceived control on mental health. Firstly, risk perception positively predicted perceived stress (β = 0.13, *SE* = 0.02, *p* < 0.01), and perceived stress positively predicted mental health (β = 0.15, *SE* = 0.02, *p* < 0.001). Secondly, when perceived stress was added, risk perception still positively predicted mental health (β = 0.13, *SE* = 0.02, *p* < 0.001). Bootstrap method was further used to test the mediating effect of perceived stress. The results showed that the 95% confidence interval does not include 0. Therefore, perceived stress partially mediated the relationship between risk perception and mental health. Hypothesis 1 was verified. Thirdly, the interaction term of perceived stress and perceived control could significantly predict mental health (β = −0.11, *SE* = 0.02, *p* < 0.001). Thus, these results suggested that the relationship between perceived stress and mental health was moderated by perceived control, that is, the influence of perceived control on mental health of college students was a moderated mediating effect. Hypothesis 2 was verified. Additionally, gender affected perceived stress (β = −0.21, *SE* = 0.07, *p* < 0.001), but not mental health (*p* > 0.05).To further illustrate how perceived control regulates the relationship between perceived stress and mental health, we took the one standard deviation above and below the mean of perceived control to draw the interaction effect graph (see Figure 2). The simple slope test (57) showed that with low perceived control (i.e., one standard deviation below the mean), perceived stress had a significant positive predictive effect on mental health (*simple slope* = 0.26, *SE* = 0.03, *p* < 0.01), while with high perceived control (i.e., one standard deviation above the mean), perceived stress could not significantly predict mental health (*simple slope* = 0.04, *SE* = 0.03, *p* = 0.21).

**TABLE 2:** Path analysis results.

	Model 1 (Perceived stress)			Model 2 (Mental health)		
		
	β (*SE*)	*t*	95%CI	β (*SE*)	*t*	95%CI
Gender	−0.21 (0.07)	−3.15***	(−0.35, −0.08)	0.08 (0.06)	1.31	(−0.04, 0.20)
Risk perception	0.13 (0.02)	5.49**	(0.08, 0.17)	0.13 (0.02)	6.08***	(0.09, 0.17)
Perceived stress				0.15 (0.02)	7.26***	(0.11, 0.19)
Perceived control				−0.35 (0.02)	−16.18***	(−0.39, −0.31)
Perceived stress* Perceived control				−0.11 (0.02)	−5.22***	(−0.16, −0.07)
*F*	21.42***			110.72***		
*R* ^2^	0.02			0.23		

N = 1,856. **p < 0.01, ***P < 0.001.

## Discussion

We examined the effect of risk perception on the mental health of college students and the role of perceived stress. In addition, we explored the effect of perceived control in the relationship between perceived stress on the mental health. Our findings indicated that college Students’ risk perception had a significant predictive effect on their mental health, and perceived stress partially mediated the relationship between risk perception on the mental health. Furthermore, the relationship between perceived stress and mental health was moderated by perceived control.

### The effect of risk perception on mental health

The results revealed that risk perception has a positive predictive effect on college Students’ mental health, that is, the higher the risk perception, the worse the mental health, which is consistent with previous research results (1, 58). This result also provided evidence for the main effect principle of the risk resilience model, which stated that risk factors and unfavorable circumstance exacerbate unfavorable outcomes (9). The negative life event of the COVID-19 epidemic has severely disrupted the normal life of college students. Due to the long incubation period, rapid contagion rate, the potential for lethality, and the lack of pharmacological interventions, this undoubtedly affects risk perceptions in some ways and leads to negative mental health outcomes (1, 59). In addition, college students may use social media more frequently than other groups (60). The social amplification of risk framework believes that crisis events interact with public psychology, social organization and social culture, and then amplify or reduce people’s risk perception of the events (61, 62). One study had found through the case analysis method that consumers’ trust crisis in the brand after accepting exaggerated information from mass media was higher than that caused by direct consumption experience (63). Huynh (64) also found that frequent use of social media was associated with higher risk perceptions of COVID-19. High-risk perception of the epidemic can easily lead to anxiety and depression, and affect mental health.

### The mediating effect of perceived stress

More importantly, the findings showed that perceived stress partially mediates the relationship between risk perception and mental health. Research showed that risk perception was significantly correlated with perceived stress, and risk perception of environmental threats often translated into perceived stress (1, 65). Excessive risk perception of the epidemic may increase the individuals’ perceptions of insecurity and uncertainty about their current situation, resulting in increased perceived stress. In addition, the higher the perceived stress, the worse the mental health of college students (25, 26). The college student population is one of the most susceptible groups in the epidemic and faces considerable stress in terms of health, academics, economy, and interpersonal relationships, which may be an important reason for the poor mental health of college students during the pandemic (6, 7). Overall, this result showed that risk perception of the pandemic triggered perceptions of stress, which in turn affected the mental health of college students, suggesting that perceived stress was a potential mechanism to explain the effects of risk perception on mental health in college students. This is consistent with the views of the stress process theory and the risk resilience model. Stress process theory argues that negative life events can lead to adverse changes in people’s lives that exacerbate the level of perceived stress, and social experiences (negative life events) translate into distinct health outcomes through exposure to stress (20). One risk factor can increase the likelihood of exposure to another risk factor, thereby increasing the likelihood of college students being affected by multiple risk factors. The risk resilience model also points out that extrinsic risk factors and adverse circumstances can exacerbate adverse outcomes (e.g., fear, anxiety, depression), and different types of risk factors can be calculated cumulatively. The college student population itself was not fully mature enough to deal with crises, and when they exposed to negative life events (e.g., the COVID-19 pandemic), both fears about the current situation and fear of adverse future consequences would add up to a huge psychological stress, which in turn exacerbates negative mental health states. On the other hand, previous research had demonstrated that positive messages, such as government proactive preventive measures, could reduce the level of risk perception (66). Other stress reduction strategies such as physical activity, mindfulness training, and a healthy diet could reduce perceived stress (67). These training methods may be able to improve the mental health of college students by reducing risk perception and perceived stress.

### The moderating effect of perceived control

As stated in Hypothesis 2, perceived control moderated the relationship between perceived stress and college Students’ mental health. Under low perceived control condition, perceived stress effected mental health, and conversely, perceived stress had no significant effect on mental health under high perceived control condition. This result could be explained by the stress transactional model. The theory states that when individuals perceive stress, they will conduct cognitive appraisal. The first stage is the primary cognitive appraisal, in which the individual judges the severity of the stress internally. If the stress is considered to be threatening, a secondary cognitive appraisal is performed, in which the individual assesses own resources to deal with the stress. Perceived control is a critical factor in the secondary cognitive appraisal process and is an important personal (coping) resource (47, 68). When perceived control resource is insufficient to deal with external threats, perceived stress may lead to emotional distress and mental health problems, whereas perceived stress does not affect mental health (69). Individuals with high perceived control believe that changes in the environment depend on their own actions, efforts, and choices (70), and employ more problem-focused coping than emotion-focused coping (71). Research had found that perceived control was related to perceptual stress (72). For example, Bollini et al. (73) found that perceived control reduced the increase in perceived stress produced by exposure to aversive stimulus, and perceived control over the stress attenuated cortisol secretion. Perceived control can reduce perceived stress, possibly because perceived control is associated with a strong sense of self-efficacy and the ability to adapt to society, and it encourages individuals to actively deal with and solve problems, thereby reducing the individual’s perceived stress (37). Additionally, many studies have found that perceived control was significantly associated with mental health, and individuals with high perceived control have better mental health (39). This study supported the risk resilience model. The main effect principle of the risk resilience model suggests that risk and adversity exacerbate the propensity for adverse outcomes. Whereas the compensatory effect states that enough positive internal and/or external assets could offset the adverse outcomes suffered from the negative effects of risk factors. Therefore, people with sufficient assets are less exposed to risk, and have better results than those with the same risk level but insufficient assets. This also explains that in this study, compared with the low perceived control condition, the effect of perceived stress on mental health was smaller or even disappeared under the high perceived control condition. In conclusion, this study showed that perceived control could serve as a protective factor to buffer against the adverse effects of the COVID-19 pandemic on college Students’ mental health. However, college students with low levels of perceived control may be affected by perceived stress and experience adverse psychological symptoms. Therefore, we need to provide more psychological assistance to those college students with poor perceived control to protect their mental health and prevent them from developing more mental health problems.

### Limitations and future research

The present study has several limitations that should be noted. Firstly, this study adopted a cross-sectional design to explore the relationship among risk perception, perceived stress, perceived control, and mental health of college students. However, the cross-sectional design has its limitations. Future studies could use other approaches, such as longitudinal designs or experimental studies, to continue to explore this question in greater depth. Secondly, the study used a convenient sampling method to collect data. The sample size varies greatly in different provinces/regions and was not balanced by gender. Therefore, the interpretation of the data results should be cautious. Future studies could explore the relationship among these variables based on a more balanced sample from multiple regions. Finally, due to the use of an online survey, all participants’ data could only be assessed using self-report instruments. There may be specific errors in the self-report method, such as social desirability bias and response sets, in which participants respond to questions according to socially accepted standards rather than true intentions or their own specific behavior patterns. In future research, structured interviews and evaluations by others (such as teachers and friends) could be used to assess the psychological state of college students more accurately.

## Conclusion

The present study showed that risk perception of COVID-19 was significantly correlated with mental health. Furthermore, perceived stress mediated the relationship between risk perception and mental health, and perceived control moderated the relationship between perceived stress and mental health among Chinese college students during the COVID-19 pandemic.

## Data availability statement

The original contributions presented in this study are included in the article/Supplementary material, further inquiries can be directed to the corresponding author.

## Ethics statement

The studies involving human participants were reviewed and approved by the School of psychology, Northwest Normal University. The patients/participants provided their written informed consent to participate in this study.

## Author contributions

LL: conceptualization, investigation, data collection, formal analysis, writing – original draft, and writing – review and editing. HC: conceptualization, resources, investigation, data collection, methodology, visualization, supervision, and writing – review and editing. LY: conceptualization, funding acquisition, investigation, data collection, and project administration. CY: conceptualization, resources, investigation, data collection, and visualization. XW: conceptualization, resources, investigation, and data collection. YM: conceptualization, investigation, and data collection. All authors contributed to the article and approved the submitted version.
