# Supplementary material for: Risk perception and mental health among college students in China during the COVID-19 pandemic: A moderated mediation model
Source: Front Psychiatry. 2022 Aug 1;13:955093. doi: 10.3389/fpsyt.2022.955093 (PMC9376247; doi:10.3389/fpsyt.2022.955093)
Supplement: Supplementary file 1 [file Table_1.DOCX]

**Power Analysis**

To calculate statistical power and test the estimator performance for moderated mediation model with current sample size (N = 1856), a series of Carlo power analyses with 1,0000 replications was conducted in Mplus version 8.3 to determine the power for α=0.05. The cross-paths between risk perception, perceived stress, perceived control, and mental health were set to 0.10 in the simulation. This effect size was typically considered as the smallest effect relevant for interpretation (e.g., Funder & Ozer, 2019). Results showed that for the moderated mediation model with N =1856, if path coefficients between variables were set to be r = 0.1, statistical power was high (all above 0.95, see table 1).

Table 1 Power Analysis

| **Path** | **Estimated Effect** | **Power** |
| --- | --- | --- |
| Perceived Stress on Risk Perception | 0.100 | 0.991 |
| Mental Health on Risk Perception | 0.100 | 0.990 |
| Mental Health on Perceived Stress | 0.100 | 0.993 |
| Mental Health on Perceived Control | 0.100 | 0.992 |
| Mental Health on Perceived Stress* Perceived Control | 0.100 | 0.991 |
| Risk Perception with Perceived Control | 0.100 | 0.990 |
| Risk Perception with Perceived Stress* Perceived Control | 0.100 | 0.991 |
| Perceived Control with Perceived Stress* Perceived Control | 0.100 | 0.992 |
